# Crystal structure of μ-peroxido-κ^4^ *O* ^1^,*O* ^2^:*O* ^1′^,*O* ^2′^-bis­[(nitrato-κ*O*)(2,2′:6′,2′′-terpyridine-κ^3^ *N*,*N*′,*N*′′)dioxidouranium(VI)]

**DOI:** 10.1107/S2056989015007987

**Published:** 2015-04-25

**Authors:** Takeshi Kawasaki, Takafumi Kitazawa

**Affiliations:** a Department of Chemistry, Faculty of Science, Toho University, 2-2-1 Miyama, Funabashi, Chiba 274-8510, Japan; b Research Center for Materials with Integrated Properties, Toho University, Miyama, Funabashi, Chiba 274-8510, Japan

**Keywords:** crystal structure, uranium(VI) complex, dimer, peroxide, 2,2′:6′,2′′-terpyridine, uran­yl(VI) ion

## Abstract

In the title dimeric complex, [{UO_2_(NO_3_)(C_15_H_11_N_3_)}_2_O_2_], a peroxide ion bridges the two uran­yl(VI) [O=U=O]^2+^ ions. The O—O bond length of the peroxide is 1.485 (6) Å and the mid-point of this bond is located at the inversion centre of the dimer. The U atom exhibits a distorted hexa­gonal–bipyramidal coordination geometry with two uran­yl(VI) O atoms occupying the axial positions and one O atom of the monodentate nitrate ion, both O atoms of the peroxide ion and the three N atoms of the chelating tridentate 2,2′:6′,2′′-terpyridine (terpy) ligand in the equatorial positions. Two of the N atoms of the terpy ligand lie above and below the mean plane containing the equatorial ligand atoms and the U atom [deviations from the mean plane: maximum 0.500 (2), minimum −0.472 (2) and r.m.s. = 0.2910 Å]. The dihedral angle between the terpy ligand and the mean plane is 35.61 (7)°. The bond lengths around the U atom decrease in the order U—N > U—O_nitrate_ > U—O_peroxo_ > U=O. The dimeric complexes pack in a three-dimensional network held together by weak π–π inter­actions [centroid–centroid distance = 3.659 (3) Å] between pyridyl rings of the terpy ligands in neighbouring dimers, together with inter­molecular C—H⋯O and C—H⋯π inter­actions. Weak intra­molecular C—H⋯O inter­actions are also observed.

## Related literature   

For the structures of uran­yl(VI) complexes with terpy, see: Berthet *et al.* (2004 ▸). For the structures of uran­yl(VI) μ-κ^2^:κ^2^-peroxide complexes, see:Charushnikova *et al.* (2001 ▸); Goff *et al.* (2008 ▸); John *et al.* (2004 ▸); Sigmon *et al.* (2009 ▸); Takao & Ikeda (2010 ▸). For the structures of a uran­yl(VI) complex with terpy and a uran­yl(VI) μ-κ^2^:κ^2^-peroxide complex, see: Charushnikova & Den Auwer (2004 ▸).
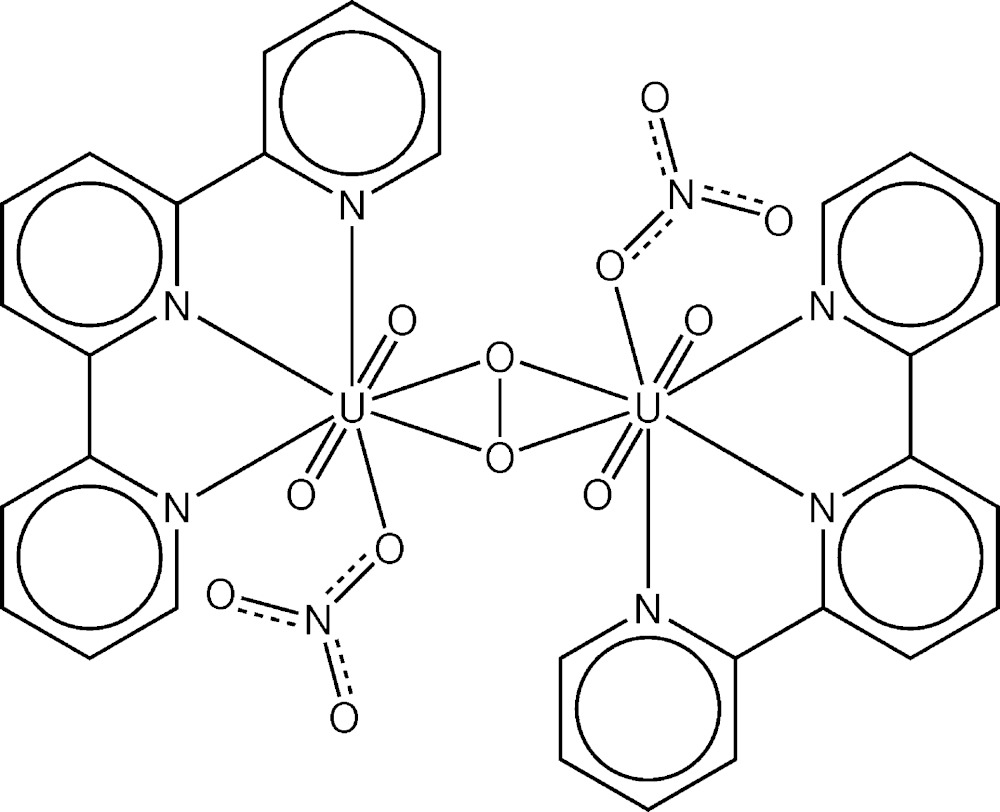


## Experimental   

### Crystal data  

[U_2_(NO_3_)_2_(O_2_)O_4_(C_15_H_11_N_3_)_2_]
*M* *_r_* = 1162.62
Monoclinic, 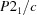

*a* = 13.4924 (11) Å
*b* = 10.2791 (8) Å
*c* = 12.6977 (10) Å
β = 114.691 (1)°
*V* = 1600.0 (2) Å^3^
*Z* = 2
Mo *K*α radiation
μ = 10.19 mm^−1^
*T* = 90 K
0.28 × 0.14 × 0.06 mm

### Data collection  

Bruker APEXII CCD area-detector diffractometer
Absorption correction: analytical (*XPREP*; Bruker, 2007 ▸) *T*_min_ = 0.163, *T*_max_ = 0.580
11692 measured reflections
4695 independent reflections
3636 reflections with *I* > 2σ(*I*)
*R*_int_ = 0.063

### Refinement  

*R*[*F* ^2^ > 2σ(*F* ^2^)] = 0.030
*wR*(*F* ^2^) = 0.058
*S* = 0.89
4695 reflections
235 parameters
H-atom parameters constrained
Δρ_max_ = 2.24 e Å^−3^
Δρ_min_ = −1.57 e Å^−3^

### 

Data collection: *APEX2* (Bruker, 2007 ▸); cell refinement: *SAINT* (Bruker, 2007 ▸); data reduction: *SAINT*; program(s) used to solve structure: *SHELXS97* (Sheldrick, 2008 ▸); program(s) used to refine structure: *SHELXL97* (Sheldrick, 2008 ▸); molecular graphics: *SHELXTL* (Sheldrick, 2008 ▸); software used to prepare material for publication: *SHELXTL* and *PLATON* (Spek, 2009 ▸).

## Supplementary Material

Crystal structure: contains datablock(s) I, global. DOI: 10.1107/S2056989015007987/cq2015sup1.cif

Structure factors: contains datablock(s) I. DOI: 10.1107/S2056989015007987/cq2015Isup2.hkl

**Fig. 1.**
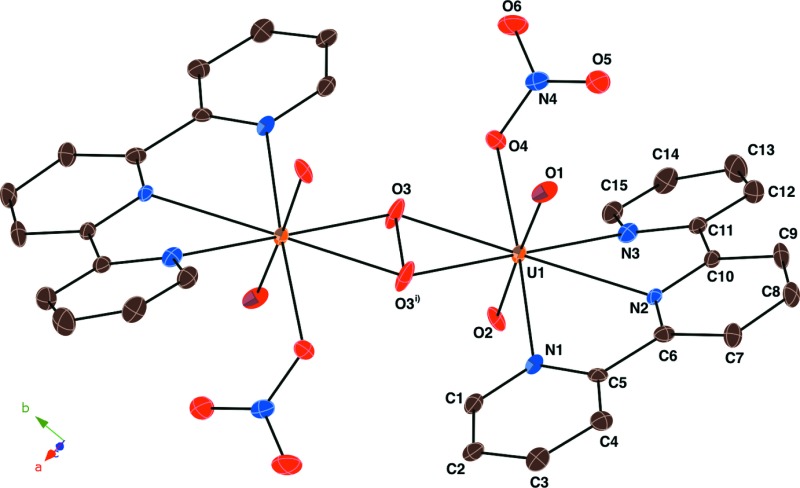
Structure of the dimer [{UO2(NO3)(C15H11N3)}2O2]. Displacement ellipsoids are drawn at the 50% probability level. H atoms are omitted for clarity. [Symmetry code: (i) -x + 1, -y + 1, -z + 1]

2 3 15 11 3 2 2 x y z . DOI: 10.1107/S2056989015007987/cq2015fig1.tif
Structure of the dimer [{UO_2_(NO_3_)(C_15_H_11_N_3_)}_2_O_2_]. Displacement ellipsoids are drawn at the 50% probability level. H atoms are omitted for clarity. [Symmetry code: (i) −*x* + 1, −*y* + 1, −*z* + 1]

**Fig. 2.**
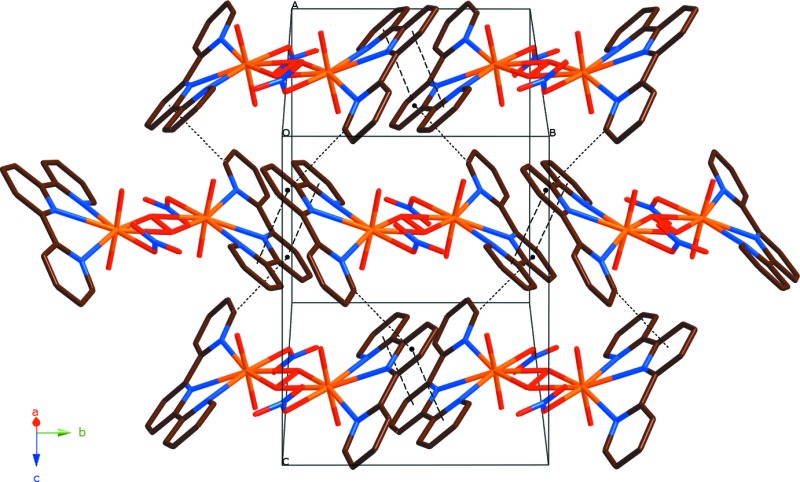
Packing diagram of [{UO2(NO3)(C15H11N3)}2O2]. Dashed lines and dotted lines are π–π and C—H···π interactions, respectively.

2 3 15 11 3 2 2 . DOI: 10.1107/S2056989015007987/cq2015fig2.tif
Packing diagram of [{UO_2_(NO_3_)(C_15_H_11_N_3_)}_2_O_2_]. Dashed lines and dotted lines are π–π and C—H⋯π inter­actions, respectively.

CCDC reference: 1061056

Additional supporting information:  crystallographic information; 3D view; checkCIF report

## Figures and Tables

**Table:** 

U1O1	1.777(3)
U1O2	1.775(3)
U1O3	2.340(3)
U1O3^i^	2.325(3)
U1O4	2.479(3)
U1N1	2.574(3)
U1N2	2.634(3)
U1N3	2.593(3)
O3O3^i^	1.485(6)
O4N4	1.295(5)
O5N4	1.232(5)
O6N4	1.240(4)

**Table:** 

O1U1O2	177.31(13)
O1U1O3	91.64(14)
O1U1O3^i^	90.58(14)
O1U1O4	85.85(12)
O3^i^U1O3	37.12(13)
O3U1O4	66.75(10)
O1U1N1	89.42(13)
O3^i^U1N1	71.44(10)
O1U1N2	76.01(12)
O1U1N3	100.67(13)
O4U1N3	70.03(11)
N1U1N2	61.29(11)
N2U1N3	60.44(11)

Symmetry code: (i) 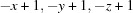.

**Table 2:** Hydrogen-bond geometry (, ) *Cg*2 is the centroid of the C6C10/N2 ring.

*D*H*A*	*D*H	H*A*	*D* *A*	*D*H*A*
C1H1O3^i^	0.95	2.28	2.773(6)	112
C1H1O4^i^	0.95	2.59	3.225(5)	125
C2H2O6^i^	0.95	2.59	3.357(6)	137
C3H3O1^ii^	0.95	2.58	3.176(6)	121
C4H4O1^ii^	0.95	2.55	3.162(6)	122
C12H12O5^iii^	0.95	2.32	3.256(6)	169
C14H14O6^iv^	0.95	2.48	3.246(7)	138
C15H15*Cg*2^v^	0.95	2.62	3.512(5)	157

Symmetry codes: (i) 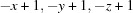; (ii) 
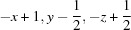
; (iii) 
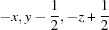
; (iv) 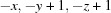; (v) 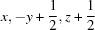.

## supplementary crystallographic information

### S1. Experimental

10 ml of a methanolic solution containing 0.5 mmol of terpy was added to 10 ml of a methanolic solution containing 0.5 mmol of UO_2_(NO_3_)_2_·6H_2_O contained in a glass sample vial. The vial w as sealed with a lid and kept in sunlight at room temperature. Yellow crystals grew after one day. The crystal structure of the yellow material has not yet been determined. After about two months, orange crystals of the title complex were obtained.

### S2. Refinement

All H atoms were placed at calculated positions, with C(CH)—H = 0.95 Å and allowed to ride on the parent atoms, with *U*_iso_(H) = 1.2*U*_eq_(C). The (1 0 0) reflection, affected by the beamstop, was omitted from the final refinement.

### Crystal data

**Table:**

[U_2_(NO_3_)_2_(O_2_)O_4_(C_15_H_11_N_3_)_2_]	*F*(000) = 1076
*M**_r_* = 1162.62	*D*_x_ = 2.413 Mg m^−^^3^
Monoclinic, *P*2_1_/*c*	Mo *K*α radiation, λ = 0.71073 Å
Hall symbol: -P 2ybc	Cell parameters from 4370 reflections
*a* = 13.4924 (11) Å	θ = 2.6–30.1°
*b* = 10.2791 (8) Å	µ = 10.19 mm^−^^1^
*c* = 12.6977 (10) Å	*T* = 90 K
β = 114.691 (1)°	Plate, orange
*V* = 1600.0 (2) Å^3^	0.28 × 0.14 × 0.06 mm
*Z* = 2	

### Data collection

**Table:**

Bruker APEXII CCD area-detector diffractometer	4695 independent reflections
Radiation source: fine-focus sealed tube	3636 reflections with *I* > 2σ(*I*)
Graphite monochromator	*R*_int_ = 0.063
Detector resolution: 8.333 pixels mm^-1^	θ_max_ = 31.0°, θ_min_ = 2.6°
phi and ω scans	*h* = −19→17
Absorption correction: analytical (*XPREP*; Bruker, 2007)	*k* = −14→5
*T*_min_ = 0.163, *T*_max_ = 0.580	*l* = −18→18
11692 measured reflections	

### Refinement

**Table:**

Refinement on *F*^2^	Primary atom site location: structure-invariant direct methods
Least-squares matrix: full	Secondary atom site location: difference Fourier map
*R*[*F*^2^ > 2σ(*F*^2^)] = 0.030	Hydrogen site location: inferred from neighbouring sites
*wR*(*F*^2^) = 0.058	H-atom parameters constrained
*S* = 0.89	*w* = 1/[σ^2^(*F*_o_^2^) + (0.0123*P*)^2^] where *P* = (*F*_o_^2^ + 2*F*_c_^2^)/3
4695 reflections	(Δ/σ)_max_ = 0.001
235 parameters	Δρ_max_ = 2.24 e Å^−^^3^
0 restraints	Δρ_min_ = −1.57 e Å^−^^3^

### Special details

**Table:**

Experimental. face-indexed absorption correction carried out with *XPREP* (Bruker, 2007)
Geometry. All e.s.d.'s (except the e.s.d. in the dihedral angle between two l.s. planes) are estimated using the full covariance matrix. The cell e.s.d.'s are taken into account individually in the estimation of e.s.d.'s in distances, angles and torsion angles; correlations between e.s.d.'s in cell parameters are only used when they are defined by crystal symmetry. An approximate (isotropic) treatment of cell e.s.d.'s is used for estimating e.s.d.'s involving l.s. planes.
Refinement. Refinement of *F*^2^ against ALL reflections. The weighted *R*-factor *wR* and goodness of fit *S* are based on *F*^2^, conventional *R*-factors *R* are based on *F*, with *F* set to zero for negative *F*^2^. The threshold expression of *F*^2^ > σ(*F*^2^) is used only for calculating *R*-factors(gt) *etc*. and is not relevant to the choice of reflections for refinement. *R*-factors based on *F*^2^ are statistically about twice as large as those based on *F*, and *R*- factors based on ALL data will be even larger.

### Fractional atomic coordinates and isotropic or equivalent isotropic displacement parameters (Å^2^)

**Table:**

	*x*	*y*	*z*	*U*_iso_*/*U*_eq_	
U1	0.387048 (13)	0.333645 (14)	0.466857 (14)	0.00993 (5)	
O1	0.3311 (3)	0.3633 (3)	0.3151 (3)	0.0176 (7)	
O2	0.4441 (3)	0.2963 (3)	0.6171 (3)	0.0168 (7)	
O3	0.4596 (3)	0.5439 (3)	0.5073 (4)	0.0306 (10)	
O4	0.2546 (2)	0.4979 (3)	0.4721 (3)	0.0152 (6)	
O5	0.1152 (3)	0.4243 (3)	0.3199 (3)	0.0242 (8)	
O6	0.1038 (3)	0.6064 (3)	0.4006 (3)	0.0283 (9)	
N1	0.5298 (3)	0.1823 (3)	0.4461 (3)	0.0126 (7)	
N2	0.3249 (3)	0.1020 (3)	0.3740 (3)	0.0097 (7)	
N3	0.2240 (3)	0.2190 (3)	0.4857 (3)	0.0129 (7)	
N4	0.1554 (3)	0.5090 (4)	0.3945 (3)	0.0165 (8)	
C1	0.6361 (4)	0.2172 (4)	0.4933 (4)	0.0142 (9)	
H1	0.6585	0.2851	0.5491	0.017*	
C2	0.7141 (4)	0.1591 (4)	0.4644 (4)	0.0147 (9)	
H2	0.7882	0.1858	0.5006	0.018*	
C3	0.6820 (4)	0.0616 (4)	0.3821 (4)	0.0157 (9)	
H3	0.7329	0.0226	0.3580	0.019*	
C4	0.5733 (3)	0.0215 (4)	0.3351 (4)	0.0146 (9)	
H4	0.5494	−0.0468	0.2797	0.018*	
C5	0.5008 (3)	0.0823 (4)	0.3700 (4)	0.0109 (8)	
C6	0.3841 (3)	0.0422 (4)	0.3258 (4)	0.0116 (8)	
C7	0.3392 (4)	−0.0538 (4)	0.2412 (4)	0.0149 (9)	
H7	0.3831	−0.0977	0.2103	0.018*	
C8	0.2299 (4)	−0.0839 (4)	0.2032 (4)	0.0163 (9)	
H8	0.1971	−0.1464	0.1436	0.020*	
C9	0.1686 (4)	−0.0219 (4)	0.2529 (4)	0.0164 (9)	
H9	0.0934	−0.0415	0.2281	0.020*	
C10	0.2192 (3)	0.0695 (4)	0.3396 (4)	0.0108 (8)	
C11	0.1638 (4)	0.1318 (4)	0.4050 (4)	0.0118 (8)	
C12	0.0579 (4)	0.0988 (4)	0.3906 (4)	0.0173 (10)	
H12	0.0148	0.0396	0.3318	0.021*	
C13	0.0181 (4)	0.1539 (5)	0.4633 (5)	0.0229 (11)	
H13	−0.0543	0.1352	0.4530	0.027*	
C14	0.0819 (4)	0.2362 (4)	0.5515 (4)	0.0193 (10)	
H14	0.0564	0.2702	0.6052	0.023*	
C15	0.1850 (4)	0.2679 (4)	0.5589 (4)	0.0156 (9)	
H15	0.2293	0.3261	0.6179	0.019*	

### Atomic displacement parameters (Å^2^)

**Table:**

	*U*^11^	*U*^22^	*U*^33^	*U*^12^	*U*^13^	*U*^23^
U1	0.01075 (8)	0.00727 (7)	0.01268 (8)	−0.00116 (7)	0.00578 (6)	−0.00132 (7)
O1	0.0249 (18)	0.0142 (15)	0.0175 (17)	0.0044 (14)	0.0126 (15)	0.0039 (13)
O2	0.0163 (16)	0.0199 (15)	0.0080 (16)	0.0007 (14)	−0.0011 (13)	−0.0061 (13)
O3	0.0182 (18)	0.0076 (14)	0.075 (3)	0.0007 (14)	0.028 (2)	−0.0085 (17)
O4	0.0111 (14)	0.0162 (14)	0.0164 (16)	0.0003 (14)	0.0038 (13)	0.0012 (14)
O5	0.0236 (18)	0.0189 (16)	0.023 (2)	0.0019 (15)	0.0024 (16)	−0.0031 (15)
O6	0.0224 (19)	0.0228 (17)	0.034 (2)	0.0124 (16)	0.0064 (17)	−0.0034 (17)
N1	0.0164 (18)	0.0082 (16)	0.0143 (19)	−0.0006 (15)	0.0076 (16)	−0.0001 (14)
N2	0.0126 (17)	0.0074 (15)	0.0074 (17)	−0.0017 (14)	0.0025 (15)	0.0016 (13)
N3	0.0180 (19)	0.0115 (16)	0.0102 (19)	0.0002 (16)	0.0069 (16)	0.0012 (14)
N4	0.0161 (18)	0.0158 (17)	0.017 (2)	0.0033 (18)	0.0066 (16)	0.0043 (17)
C1	0.018 (2)	0.0094 (18)	0.016 (2)	−0.0009 (18)	0.007 (2)	0.0004 (17)
C2	0.016 (2)	0.0102 (18)	0.019 (2)	0.0000 (19)	0.0094 (19)	0.0028 (18)
C3	0.022 (2)	0.016 (2)	0.014 (2)	0.0001 (19)	0.012 (2)	0.0025 (18)
C4	0.018 (2)	0.015 (2)	0.010 (2)	0.0022 (18)	0.0046 (18)	0.0002 (17)
C5	0.013 (2)	0.0101 (18)	0.008 (2)	0.0009 (17)	0.0025 (17)	0.0038 (16)
C6	0.014 (2)	0.0117 (19)	0.009 (2)	0.0030 (17)	0.0043 (18)	0.0006 (16)
C7	0.018 (2)	0.015 (2)	0.013 (2)	−0.0036 (19)	0.0064 (19)	−0.0046 (17)
C8	0.022 (2)	0.014 (2)	0.009 (2)	−0.0040 (19)	0.0031 (19)	−0.0054 (17)
C9	0.016 (2)	0.018 (2)	0.013 (2)	−0.0082 (19)	0.0035 (18)	−0.0031 (18)
C10	0.013 (2)	0.0084 (18)	0.009 (2)	−0.0002 (17)	0.0033 (17)	−0.0006 (16)
C11	0.014 (2)	0.0091 (18)	0.010 (2)	0.0002 (17)	0.0036 (17)	0.0012 (16)
C12	0.017 (2)	0.016 (2)	0.019 (3)	−0.0022 (19)	0.007 (2)	0.0003 (19)
C13	0.014 (2)	0.026 (2)	0.032 (3)	−0.004 (2)	0.013 (2)	−0.002 (2)
C14	0.024 (3)	0.017 (2)	0.023 (3)	−0.001 (2)	0.016 (2)	−0.0033 (19)
C15	0.021 (2)	0.013 (2)	0.013 (2)	−0.0002 (19)	0.0074 (19)	−0.0010 (17)

### Geometric parameters (Å, º)

**Table:**

U1—O1	1.777 (3)	C2—H2	0.9500
U1—O2	1.775 (3)	C3—C4	1.395 (6)
U1—O3	2.340 (3)	C3—H3	0.9500
U1—O3^i^	2.325 (3)	C4—C5	1.380 (6)
U1—O4	2.479 (3)	C4—H4	0.9500
U1—N1	2.574 (3)	C5—C6	1.492 (6)
U1—N2	2.634 (3)	C6—C7	1.397 (6)
U1—N3	2.593 (3)	C7—C8	1.381 (6)
O3—O3^i^	1.485 (6)	C7—H7	0.9500
O3—U1^i^	2.325 (3)	C8—C9	1.388 (6)
O4—N4	1.295 (5)	C8—H8	0.9500
O5—N4	1.232 (5)	C9—C10	1.391 (6)
O6—N4	1.240 (4)	C9—H9	0.9500
N1—C1	1.351 (6)	C10—C11	1.476 (6)
N1—C5	1.351 (5)	C11—C12	1.404 (6)
N2—C6	1.342 (5)	C12—C13	1.369 (6)
N2—C10	1.347 (5)	C12—H12	0.9500
N3—C15	1.343 (5)	C13—C14	1.380 (7)
N3—C11	1.348 (5)	C13—H13	0.9500
C1—C2	1.386 (6)	C14—C15	1.393 (6)
C1—H1	0.9500	C14—H14	0.9500
C2—C3	1.380 (6)	C15—H15	0.9500
			
Cg(C1–C5/N1)···Cg(C6–C10/N2)^ii^	3.659 (3)		
			
O1—U1—O2	177.31 (13)	N1—C1—H1	118.3
O1—U1—O3	91.64 (14)	C2—C1—H1	118.3
O1—U1—O3^i^	90.58 (14)	C3—C2—C1	118.7 (4)
O1—U1—O4	85.85 (12)	C3—C2—H2	120.7
O2—U1—O3	90.58 (14)	C1—C2—H2	120.7
O2—U1—O3^i^	90.28 (15)	C2—C3—C4	118.7 (4)
O2—U1—O4	96.43 (12)	C2—C3—H3	120.7
O3^i^—U1—O3	37.12 (13)	C4—C3—H3	120.7
O3—U1—O4	66.75 (10)	C5—C4—C3	119.2 (4)
O3^i^—U1—O4	103.66 (10)	C5—C4—H4	120.4
O1—U1—N1	89.42 (13)	C3—C4—H4	120.4
O2—U1—N1	88.43 (13)	N1—C5—C4	122.8 (4)
O3—U1—N1	108.56 (10)	N1—C5—C6	115.1 (4)
O3^i^—U1—N1	71.44 (10)	C4—C5—C6	122.1 (4)
O4—U1—N1	173.19 (10)	N2—C6—C7	121.7 (4)
O1—U1—N2	76.01 (12)	N2—C6—C5	115.9 (4)
O2—U1—N2	101.52 (12)	C7—C6—C5	122.4 (4)
O3—U1—N2	163.57 (12)	C8—C7—C6	118.9 (4)
O3^i^—U1—N2	130.60 (10)	C8—C7—H7	120.5
O4—U1—N2	121.97 (10)	C6—C7—H7	120.5
O1—U1—N3	100.67 (13)	C7—C8—C9	119.4 (4)
O2—U1—N3	78.83 (13)	C7—C8—H8	120.3
O3—U1—N3	133.88 (11)	C9—C8—H8	120.3
O3^i^—U1—N3	166.46 (13)	C8—C9—C10	118.7 (4)
O4—U1—N3	70.03 (11)	C8—C9—H9	120.6
N1—U1—N2	61.29 (11)	C10—C9—H9	120.6
N1—U1—N3	115.77 (11)	N2—C10—C9	121.8 (4)
N2—U1—N3	60.44 (11)	N2—C10—C11	115.3 (4)
O3^i^—O3—U1^i^	72.0 (2)	C9—C10—C11	122.8 (4)
O3^i^—O3—U1	70.9 (2)	N3—C11—C12	121.2 (4)
U1^i^—O3—U1	142.88 (13)	N3—C11—C10	115.4 (4)
N4—O4—U1	124.6 (3)	C12—C11—C10	123.3 (4)
C1—N1—C5	117.1 (4)	C13—C12—C11	118.4 (4)
C1—N1—U1	119.7 (3)	C13—C12—H12	120.8
C5—N1—U1	121.8 (3)	C11—C12—H12	120.8
C6—N2—C10	119.3 (4)	C12—C13—C14	120.9 (4)
C6—N2—U1	118.7 (3)	C12—C13—H13	119.6
C10—N2—U1	117.7 (3)	C14—C13—H13	119.6
C11—N3—C15	119.2 (4)	C13—C14—C15	117.7 (4)
C11—N3—U1	119.9 (3)	C13—C14—H14	121.2
C15—N3—U1	119.1 (3)	C15—C14—H14	121.2
O4—N4—O5	120.4 (4)	N3—C15—C14	122.4 (4)
O4—N4—O6	116.9 (4)	N3—C15—H15	118.8
O5—N4—O6	122.7 (4)	C14—C15—H15	118.8
N1—C1—C2	123.4 (4)		

Symmetry codes: (i) −*x*+1, −*y*+1, −*z*+1; (ii) −*x*+1, −*y*, −*z*+1.

### Hydrogen-bond geometry (Å, º)

Cg2 is the centroid of the C6–C10/N2 ring.

**Table:**

*D*—H···*A*	*D*—H	H···*A*	*D*···*A*	*D*—H···*A*
C1—H1···O3^i^	0.95	2.28	2.773 (6)	112
C1—H1···O4^i^	0.95	2.59	3.225 (5)	125
C2—H2···O6^i^	0.95	2.59	3.357 (6)	137
C3—H3···O1^iii^	0.95	2.58	3.176 (6)	121
C4—H4···O1^iii^	0.95	2.55	3.162 (6)	122
C12—H12···O5^iv^	0.95	2.32	3.256 (6)	169
C14—H14···O6^v^	0.95	2.48	3.246 (7)	138
C15—H15···*Cg*2^vi^	0.95	2.62	3.512 (5)	157

Symmetry codes: (i) −*x*+1, −*y*+1, −*z*+1; (iii) −*x*+1, *y*−1/2, −*z*+1/2; (iv) −*x*, *y*−1/2, −*z*+1/2; (v) −*x*, −*y*+1, −*z*+1; (vi) *x*, −*y*+1/2, *z*+1/2.

### Deviations from Least-squares plane (x,y,z in crystal coordinates).

Least-square plane: 0.0823(0.0118)x - 2.3175(0.0073)y + 11.2072(0.0058)z = 4.4537(0.0040), Rms deviation of fitted atoms = 0.2910.

**Table:**

Atom	Deviation
U1	0.0370(0.0010)
O3	0.0090(0.0041)
O3^i^	0.0555(0.0042)
O4	-0.2956(0.0023)
N1	0.1666(0.0024)
N2	-0.4723(0.0024)
N3	0.4999(0.0024)

Symmetric code: (i) -x+1,-y+1,-z+1
