# Supplementary figures and images for: Crystal structure of μ-peroxido-κ4 O 1,O 2:O 1′,O 2′-bis­[(nitrato-κO)(2,2′:6′,2′′-terpyridine-κ3 N,N′,N′′)dioxidouranium(VI)]
Source: Acta Crystallogr E Crystallogr Commun. 2015 Apr 25;71(Pt 5):m122–3. doi: 10.1107/S2056989015007987 (PMC4420076; doi:10.1107/S2056989015007987)

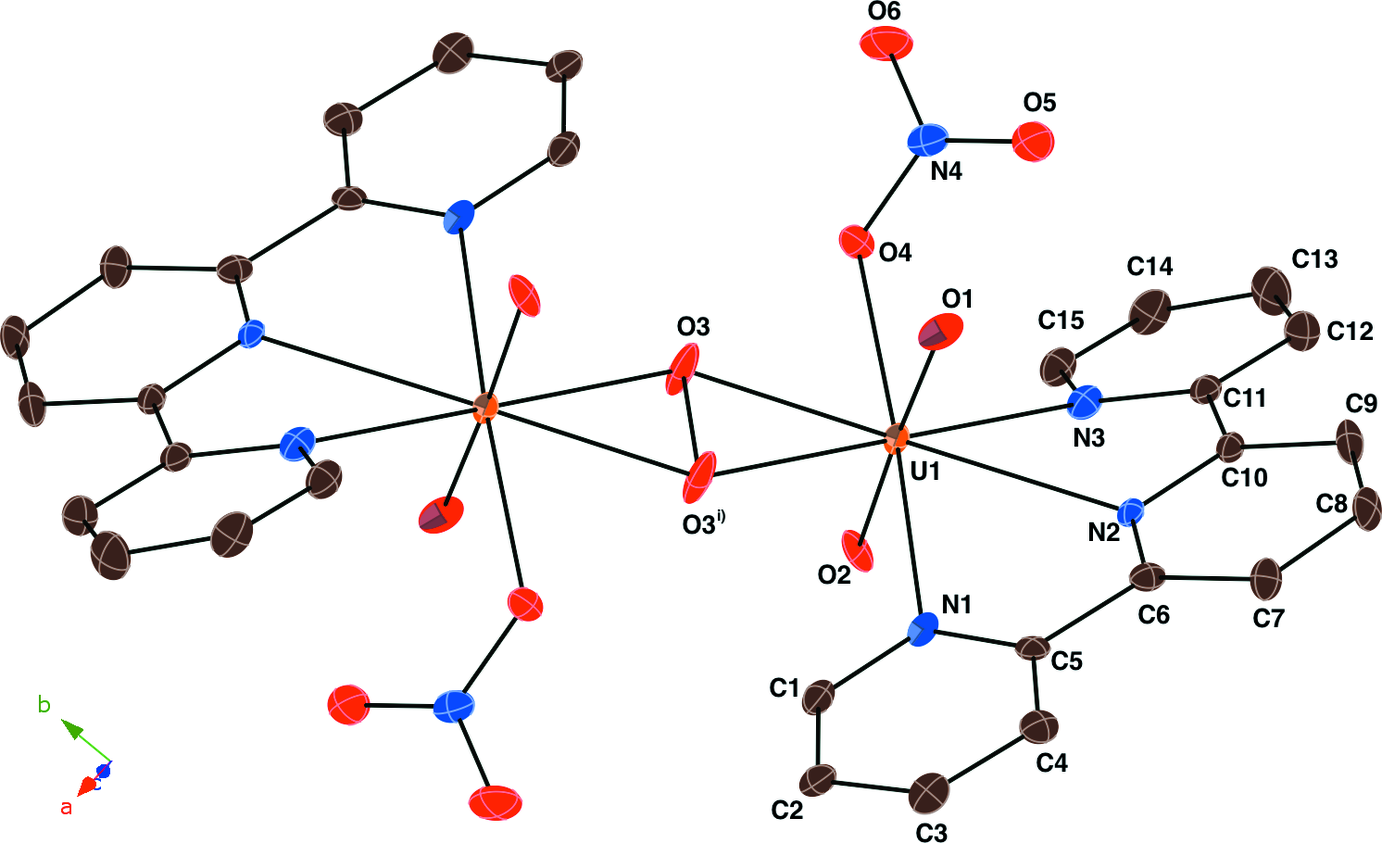

Supplement: Supplementary file 3 [file e-71-0m122-fig1.tif]

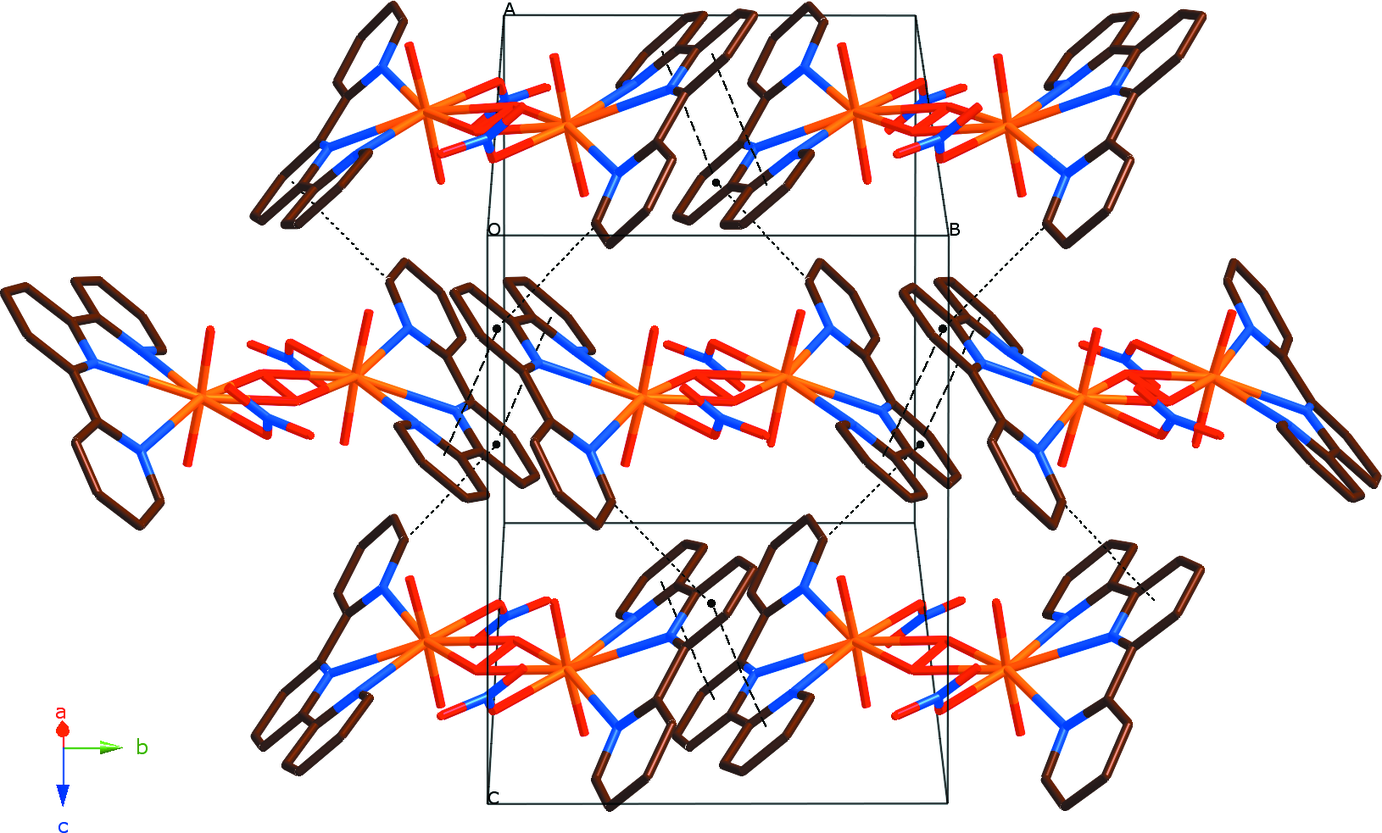

Supplement: Supplementary file 4 [file e-71-0m122-fig2.tif]
